# A case report of a triple patch reconstruction of a post-infarction ventricular septal rupture: microaxial flow pump support as a bridge to surgery

**DOI:** 10.1093/ehjcr/ytaf634

**Published:** 2025-12-03

**Authors:** Marta Medina, Susanne H Karbach, Mehmet Oezkur, Hendrik Treede, Daniel Dohle

**Affiliations:** Department of Cardiac Surgery, University Medical Center of the Johannes Gutenberg University Mainz, Langenbeckstraße 1, PH: 55131 Mainz, Germany; Department of Cardiology, University Medical Center of the Johannes Gutenberg University Mainz, Langenbeckstraße 1, PH: 55131 Mainz, Germany; Department of Cardiac Surgery, University Medical Center of the Johannes Gutenberg University Mainz, Langenbeckstraße 1, PH: 55131 Mainz, Germany; Department of Cardiac Surgery, University Medical Center of the Johannes Gutenberg University Mainz, Langenbeckstraße 1, PH: 55131 Mainz, Germany; Department of Cardiac Surgery, University Medical Center of the Johannes Gutenberg University Mainz, Langenbeckstraße 1, PH: 55131 Mainz, Germany

**Keywords:** Post-infarction ventricular septal rupture, Myocardial infarction, Cardiogenic shock, Percutaneous microaxial flow pump, Case report

## Abstract

**Background:**

Ventricular septal rupture is a rare but devastating complication of acute myocardial infarction, frequently associated with cardiogenic shock and poor prognosis. Data regarding successful treatment strategies remain limited, and operative mortality significantly decreases when surgery can be delayed by 7–14 days.

**Case Summary:**

We present the case of a 61-year-old patient diagnosed with a posterior transmural myocardial infarction complicated by a large ventricular septal rupture. Right heart catheterization showed significant right ventricular (RV) overload, with elevated right atrial pressure (20 mmHg), right ventricular end-diastolic pressure (22 mmHg), and an elevated mean pulmonary artery pressure (44 mmHg). Due to the cardiogenic shock, we reduced the left-to-right shunt and achieved right ventricular unloading using a percutaneous microaxial flow pump (mAFP) with 5.5 L/min support as a bridge to surgical repair. This hemodynamic stabilization enabled a novel approach using triple patch reconstruction for post-infarction ventricular septal rupture.

**Discussion:**

This case highlights the effectiveness of early hemodynamic support with percutaneous mAFP in stabilizing patients with acute ventricular septal rupture, facilitating delayed surgical intervention, allowing time for infarct tissue to scar. The combined use of advanced surgical techniques and mechanical support may represent a key strategy in the management of this complex condition.

Learning points

1. Ventricular septal rupture following an acute myocardial infarction is a highly lethal complication.
2. To understand the crucial role of employing mAFP with high flow to stabilize the patient and to unload the left ventricle, reducing wall stress, allowing the damaged infarcted heart tissue to heal and remodel, forming firmer scar tissue suited for surgical repair.
3. To evaluate a new technique involving triple-patch reconstruction.

## Introduction

Post-infarction ventricular septal rupture (VSR) is one of the most severe mechanical complications of acute myocardial infarction (AMI). Although rare, occurring in only 0.2–3% of AMI patients, VSR is associated with a poor prognosis and 30-day mortality rates reaching up to 54%.^1,2^ Immediate surgical repair is associated with higher mortality (54%),^3^ due to the fragility of necrotic tissue, whereas delaying surgery by at least seven days improves survival to 18.4%.^4,5^

The use of a percutaneous microaxial flow pump device with high flow (Impella 5.5®, Abiomed Inc., Danvers, Massachusetts, USA) has been proposed as a potential bridging option. By reducing shunt volume and stabilizing patients before surgery may improve outcomes.^6–8^

The prognosis after surgical repair of a post-infarct VSR remains poor, highlighting the optimal timing for surgery. This case illustrates how early hemodynamic support with the Impella-5.5 effectively allows surgical delay. Furthermore, it demonstrates a novel triple patch repair technique designed to prevent further myocardial tearing and rupture, addressing the challenges posed by the friable nature of the infarcted tissue.

## Summary figure

**Figure:**
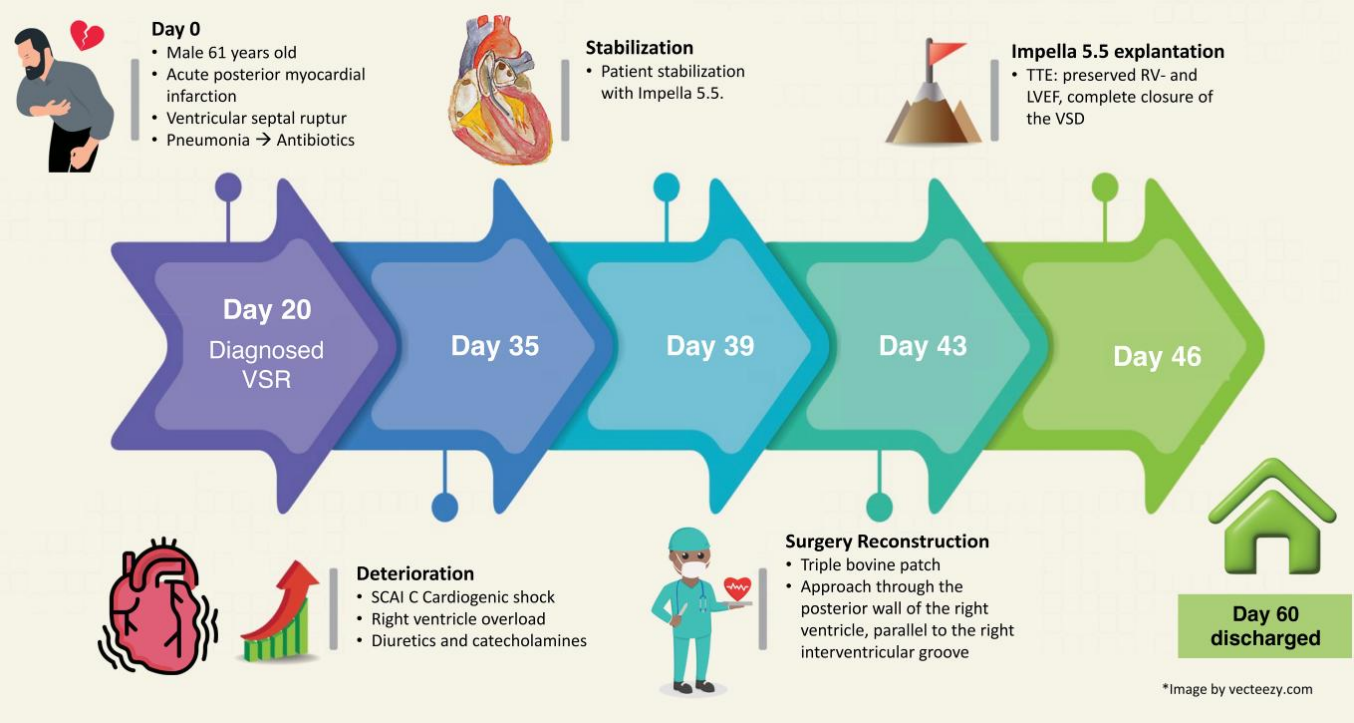


## Case presentation

**Figure 1:**
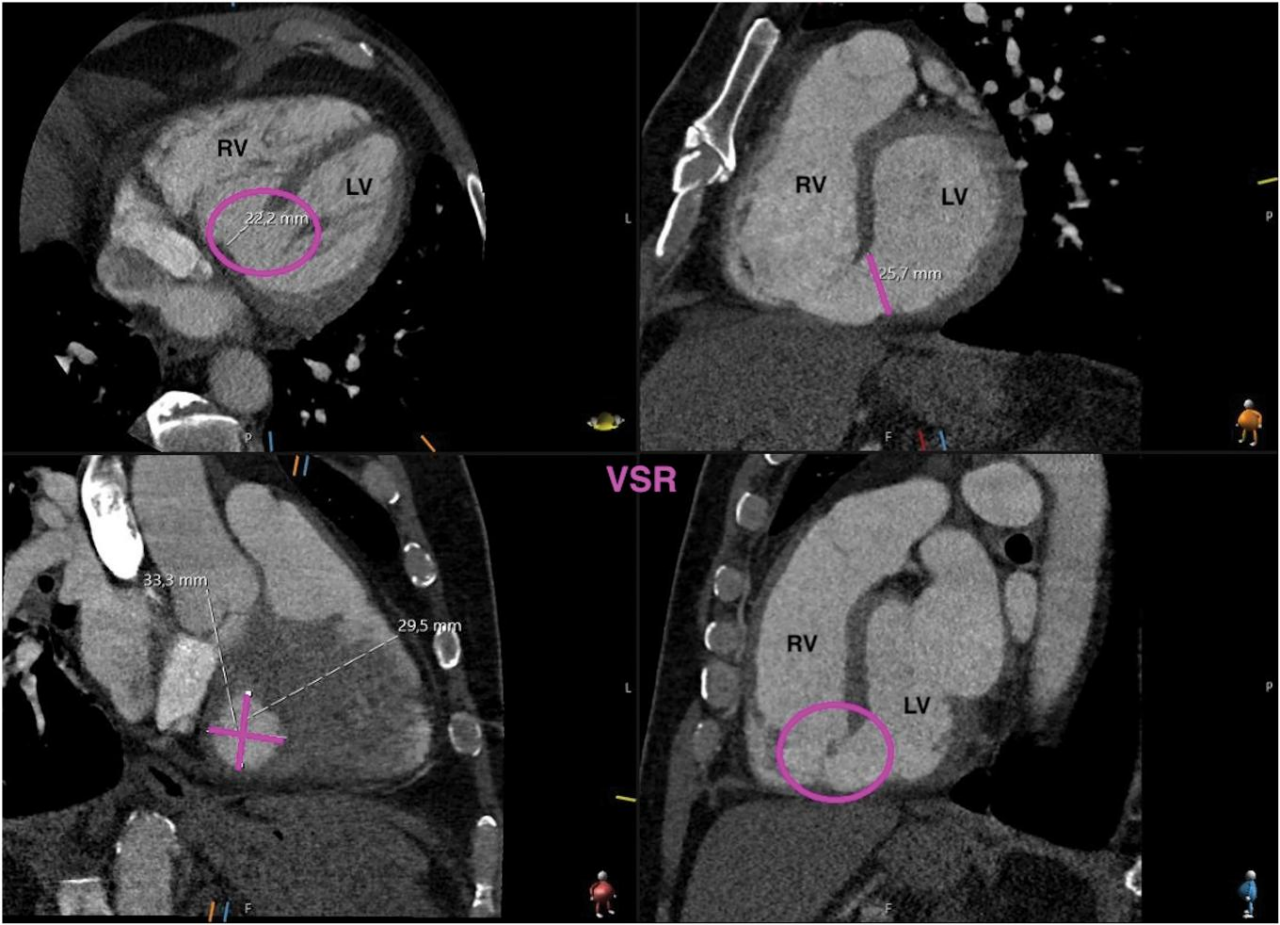
Preoperative computed tomography scan of the post-infarction ventricular septal rupture. Different views in computed tomography scan, with measurement of the VSR; VSR, ventricular septal rupture.

A 61-year-old male patient presented to our center with a complex VSR resulting from a transmural AMI of the posterior wall, following 1 month of medical recompensation at an outside hospital, where the VSR remained undiagnosed. During this period, the patient was initially treated for pneumonia with antibiotics. Due to persistent deterioration, repeat echocardiograms were eventually performed, revealing a large infero-basal VSR measuring 15 mm with a left-right shunt (see Supplementary material online, *Video S1*), preserved left ventricular ejection fraction, and signs of right heart failure. Emergent coronary angiography at our center showed an occlusion of the proximal segment of the right coronary artery. Preoperative computed tomography confirmed the VSR, measuring 30 × 33 mm (*Figure 1*).

The patient presented with cardiogenic shock (CS), making him unsuitable for immediate surgery and requiring stabilization. Despite treatment with diuretics and catecholamine as dobutamin, the therapeutic response was insufficient. As a result, he was transferred to our center for temporary mechanical circulatory support to stabilize his condition, reduce ventricular shunt volume, decrease afterload, and improve left ventricular stroke volume.

On admission, the patient was unarousable (RASS–5) despite minimal sedation with Propofol and required high doses of catecholamines (dobutamine and noradrenaline). Right heart catheterization showed significant right ventricular (RV) overload, with elevated right atrial pressure (20 mmHg), RV end-diastolic pressure (22 mmHg), and an elevated mean pulmonary artery pressure (mPAP: 44 mmHg). Therefore, after 39 days from the initial hospitalization, we implanted a microaxial flow pump (mAFP, Impella 5.5) via the left subclavian artery, providing flows up to 5.5 L/min. This intervention effectively reduced the left-to-right shunt and achieved RV unloading, resulting in a decrease of mPAP to 30 mmHg and normalization of the PCWP to 10 mmHg, as well as a systemic vascular resistance index: 2357 dyn*s*m²/cm^5^. The patient was successfully extubated after a rapid reduction in catecholamine requirements within 2 h of Impella implantation.

In the following days, transthoracic echocardiography demonstrated a gradual reduction in RV function, as well as an increase in pulmonary vascular resistance to 725 dyn*s*m²/cm^5^, despite an increase in the Impella 5.5 flow rate from 4.5 L/min to 5 L/min (P7-P9).

After achieving hemodynamic stabilization with Impella support within 7 days, surgical repair of the VSR was successfully performed. After median sternotomy, a total cardiopulmonary bypass with bicaval drainage was established.

The Impella device was initially left in place, and the aorta was cross-clamped using a soft-covered aortic clamp positioned above the Impella. Selective cardioplegia was administered through a root vent catheter with the Impella set to P1 mode to prevent significant aortic regurgitation. After achieving cardioplegic arrest, the aorta was opened, the Impella pump was dislocated and a total of 2500 mL selective antegrade cardioplegia was delivered. Cardioplegia was efficiently drained from the right atrium under total cardiopulmonary bypass.

The VSR could not be visualized through the aortic valve. Subsequently, the right atrium (RA) was opened further, but even after displacing the septal leaflet and trabecular network resection allowed only inferior exposition. Therefore, the septal leaflet was reattached, and the posterior wall exposed, revealing thinning in the area of the right interventricular posterior artery.

**Figure 2:**
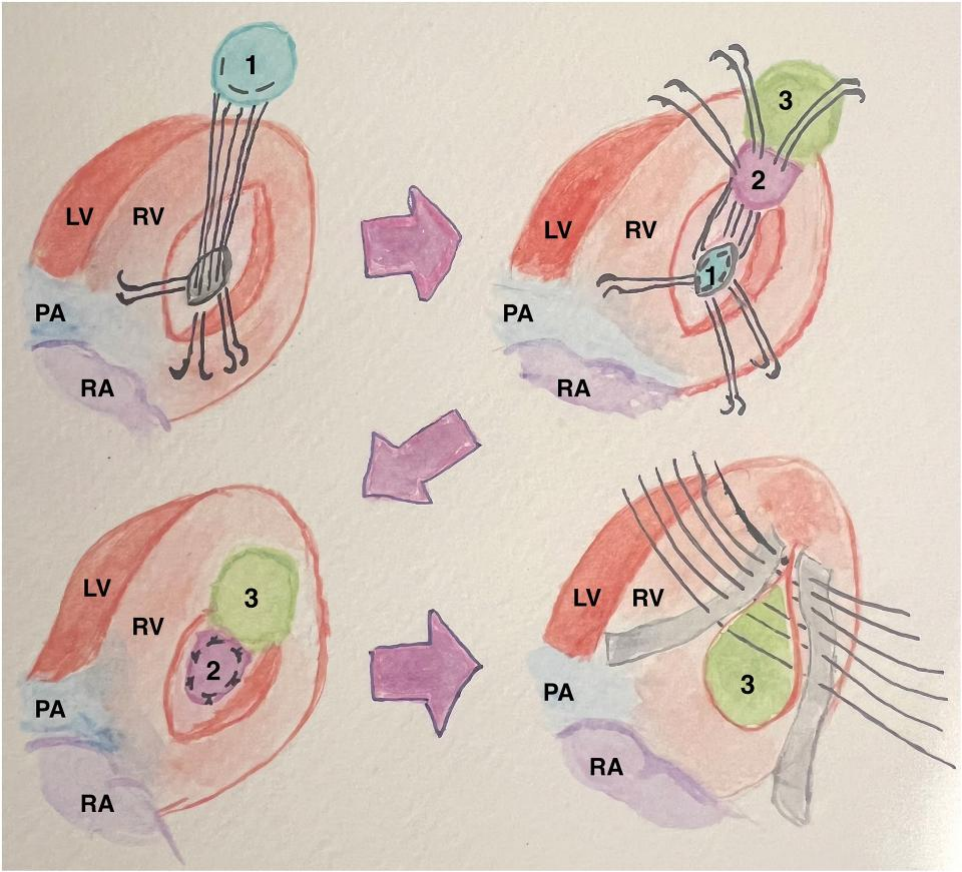
Illustration of exposed posterior wall, after longitudinal incision of the right ventricle, with exposure of the VSR: 1st. patch positioned within the LV. 2nd and 3st patch was folded to function as a double patch, which covered the septum and the free wall of the RV. Closing as a sandwich with a double-armed Teflon felt strip reinforced suture. LV, left ventricle; RV, right ventricle; VSR, ventricular septal rupture.

A 4 cm incision was performed parallel to the right interventricular groove on the posterior wall of the right ventricle, providing excellent exposure of the ventricular septum defect (VSD). A bovine pericardial patch was prepared, ∼3 × 4 cm in size. Fifteen U-shaped sutures were placed circumferentially, sequentially passing through the patch and then the VSD margins from left to right ventricular sides. The patch was then pulled through the defect into the left ventricle (LV), which generously overlapped the defect and served as a buttress for the U-Sutures (*Figure 2*).

**Figure 3:**
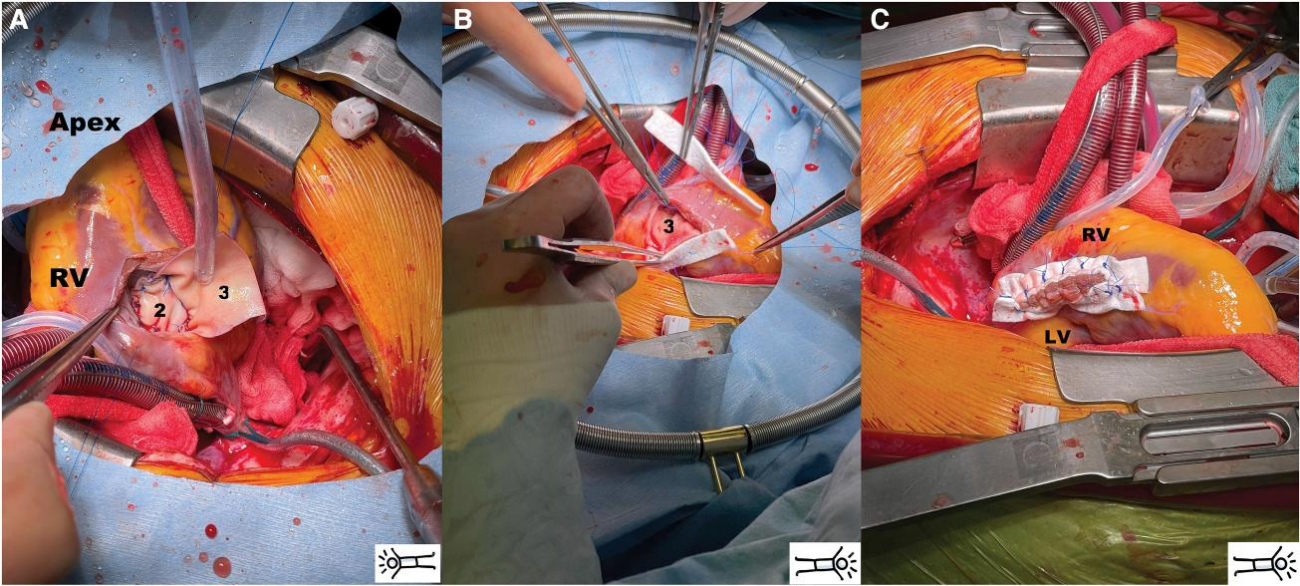
Posterior view of dislocated heart through longitudinal transinfarction incision in the RV myocardium (*A*) 2st patch positioned over the 1st patch. 2st patch is trimmed and folded to form a 3rd patch (*B*) The 3st patch is placed as a sandwich at the septum and the excess patch part is excluded to exclude the infarct area. (*C*) RV incision closed with double-armed Teflon felt strip reinforced suture.

A second pericardial patch (*Figure 3A*), twice as long as the first, was tailored. The U-sutures were geometrically aligned and passed through one-half of the patch, folding it such that its edge precisely rested at the transition from the septum to the RV free wall. After advancing the patch into position, the U-sutures were tied and cut. The second patch’s folded portion was subsequently used to reinforce the closure of the RV incision.

The ventriculotomy was closed with an initial layer of adapting mattress sutures (*Figure 3B*) followed by an over-and-over suture, both layers being tied together. The suture line was reinforced externally with a wide Teflon strip and internally with the folded portion of the second patch (*Figure 3C*).

The RV incision was sealed using TachoSil® and BioGlue® for additional hemostasis. The RA was closed, venous stasis was relieved, and the lungs were inflated. In the Trendelenburg position, the left heart and aorta were vented via the root vent, with the Impella operating in P1 mode for de-airing.

The patient was transferred to the intensive care unit in stable condition and extubated on the first post-operative day.

**Figure 4:**
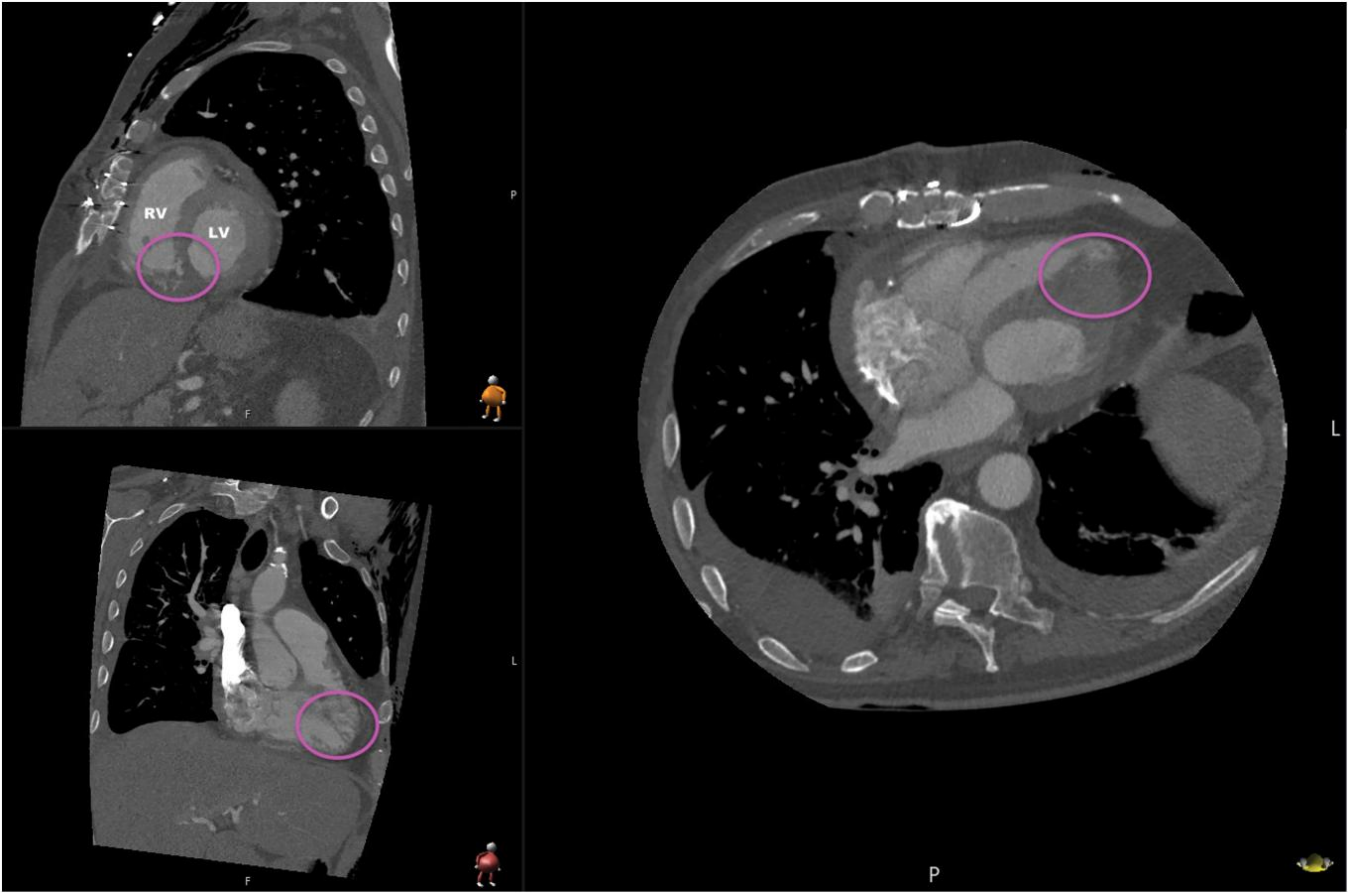
Post-operative computed tomography shows the triple patch with infarct exclusion (arrow) without residual shunt.

Post-operative echocardiography revealed well-functioning RV and LV with complete closure of the VSD and no residual shunt. Computed tomography showed good ventricular geometry and successful VSD closure (*Figure 4*). The Impella-5.5 device was uneventfully explanted 3 days after surgery. The patient was discharged 14 days post-operatively after antibiotic tazobactam treatment for pneumonia without any signs of heart failure.

## Discussion

Acute mortality of VSR remains high, compounded by the significant risk of recurrent ruptures. While delaying surgery can facilitate septal repair due to scar tissue formation, this strategy is not without risk; the rupture may expand, leading to potential fatality during the waiting period.^5,9^

Pang *et al*.^2^ identified key predictors of early mortality in post-VSD repair patients: procedure urgency, NYHA class at the time of surgery and post-operative renal failure requiring hemofiltration. Factors affecting long-term survival included NYHA class, RV-dysfunction, and LV-ejection fraction.^2^ Outcomes are particularly compromised in patients with CS and a significant burden due to left-to-right shunting.^3^ Therefore, hemodynamic stabilization, combining medical therapy and mechanical support, is essential, although the optimal timing of interventions and surgery remains uncertain.^10^

Bridging strategies, such as early veno-arterial extracorporeal membrane oxygenation (V-A ECMO) have proven effective in stabilizing patients by enhancing organ perfusion and preventing multi-organ failure,^11^ thereby enabling planned surgery.^12^ However, V-A ECMO is associated with risks, including bleeding, VSR expansion, increased afterload, and LV distention. Alternatively, Impella devices have demonstrated efficacy in reducing left-to-right shunting by lowering LV filling and improving LV total cardiac output (native output + Impella-generated output), thereby minimizing severe organ damage.^13,14^

The hemodynamic support provided by the Impella 5.5 enables prolonged preoperative stabilization, thereby optimizing patient hemodynamics and reducing surgical risk through continuous flow assistance. However, in patients with VSR, mechanical unloading carries the risk of reversing the shunt to right-to-left, leading to systemic desaturation and thromboembolic events. Early detection requires strict daily clinical and echocardiographic monitoring to confirm correct Impella positioning, with prompt flow adjustments implemented if right-to-left shunting is suspected to maintain both safety and efficacy. Therefore, left subclavian artery for Impella placement is also preferred to minimize the risk of stroke compared with right-sided access. This approach provides a more direct trajectory to the LV, potentially reducing the likelihood of embolic events and facilitating safer device insertion.

Various techniques exist for VSD closure. While percutaneous approaches can stabilize patients for delayed surgery, but they are often not feasible.^4,15^

We propose that early high-flow Impella support is a safe strategy to bridge patients with post-infarction VSR and CS to delayed surgery for myocardial recovery and operative risk mitigation. In addition, the new triple patch repair method provides a reliable approach for friable infarcted tissue, which may achieve higher success in high-risk VSR patients and underscoring the importance of a multidisciplinary staged strategy for their management.

## Lead author biography


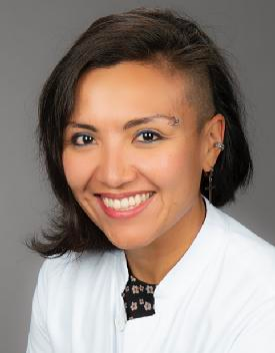


Marta Medina, MD, is a cardiac surgeon affiliated with the Department of Cardiac and Vascular Surgery at the University Medical Center of the Johannes Gutenberg University Mainz, Germany. She earned her medical degree from the Pontificia Universidad Javeriana, Bogotá, Colombia, and completed her surgical residency in Germany. Her areas of interest include heart failure, CABG surgery off-pump, and mechanical circulatory support. Dr. Medina is an active member of the DGTHG - German Society for Thoracic and Cardiovascular Surgery, EACTS – European Association for Cardio-thoracic Surgery and ISHLT – International Society for Heart & Lung Transplantation.

## Supplementary Material

ytaf634_Supplementary_Data

## Data Availability

The data underlying this article are available in the article and in its online supplementary materials.
